# Impact of WTAP in small HCC and paired adjacent non-neoplastic liver tissue on recurrence: A cohort study with external extension analysis

**DOI:** 10.3389/fcell.2022.973548

**Published:** 2022-11-07

**Authors:** Jin-Ling Duan, Min-Hua Deng, Zhi-Cheng Xiang, Jin-Long Hu, Chun-Hua Qu, Tian-Chen Zhu, Ming-Xing Xu, Jie-Wei Chen, Juan-Juan Xie, Dan Xie, Mu-Yan Cai, Mei Li, Hu Liang

**Affiliations:** ^1^ State Key Laboratory of Oncology in South China, Collaborative Innovation Center for Cancer Medicine, Sun Yat-Sen University Cancer Center, Guangzhou, China; ^2^ Department of Pathology, Sun Yat-Sen University Cancer Center, Guangzhou, China; ^3^ Department of Surgery, Sun Yat-Sen University Cancer Center, Guangzhou, China; ^4^ Department of Hepatobiliary Surgery, The Third Affiliated Hospital of Sun Yat-Sen University, Guangzhou, China; ^5^ Department of Clinical Oncology, Sun Yat-Sen University Cancer Center, Guangzhou, China

**Keywords:** WTAP, non-neoplastic liver tissue, recurrence, small hepatocellular carcinoma, external validation

## Abstract

**Background:** To evaluate prognostic value of WTAP levels in tumor and paired adjacent non-neoplastic liver tissues (PANLT) for cases of hepatitis B virus (HBV)-positive Asian small hepatocellular carcinoma (sHCC) patients who received curative partial hepatectomy.

**Method:** The investigation with two external cohorts were included. Associations between hazard risk of recurrence and continuous WTAP levels were investigated with restricted cubic spline models. Cox and inverse probability weighting models were established for survival analysis. Based on interaction effects, further stratification analysis was performed. Landmark analysis was employed to analyze cases of late recurrence. Finally, sensitivity analysis was performed to assess unmeasured confounders.

**Findings:** In an investigation cohort of 307 patients, restricted cubic spline models indicated that hazard risk of recurrence increases with elevated WTAP levels for sHCC and PANLT. However, using Cox and inverse probability weighting models, no significant differences were observed in recurrence-free survival (RFS) between groups with different WTAP levels in sHCC. Multivariate analysis showed that patients with high PANLT WTAP levels had significantly worse RFS (HR 1.567, 95% CI 1.065–2.307; *p* = 0.023). Based on the significant interaction effect between WTAP levels in sHCC and PANLT, stratification analysis revealed that recurrence risk is more pronounced in patients with high WTAP levels in both PANLT and sHCC. Landmark analysis showed that late recurrence was more likely to occur in patients with high PANLT WTAP levels (HR 2.058, 95% CI 1.113–3.805; *p* = 0.021). Moreover, the detrimental effects of elevated PANLT WTAP levels on RFS were validated with two external cohorts. Sensitivity analysis confirmed the robustness of results.

**Conclusion:** Increased PANLT WTAP expression levels independently predict high recurrence risk in HBV-positive Asian sHCC patients. Both tumor tissues and PANLT need to be considered together in future clinical practice to obtain a more comprehensive and accurate evaluation for recurrence risk.

## Introduction

Hepatocellular carcinoma (HCC) is the fourth most common worldwide cause of cancer-related death, with approximately 850,000 new cases per year (Yang et al., 2019). In China, persistent HBV infection is a major risk factor for the development of HBV-associated HCC (Llovet et al., 2016). The surveillance of HBV-positive groups contributes to early diagnosis at a relatively small tumor size (Fujiwara et al., 2018). Specifically, tumors with diameters less than 3 cm are defined as small HCC (sHCC) (Llovet et al., 2003). However, the prognosis of sHCC varies even within the same clinical stage (Feng et al., 2012; Chan et al., 2018). Meanwhile, recurrence, including early recurrence (≤2 years after treatment) and late recurrence (>2 years after treatment), is the main reason for unsatisfactory survival (EASL-EORTC clinical practice guidelines, 2012). Although the outcome of sHCC is ideal, with a median incident age of 52 in China, the 10-year recurrence-free survival (RFS) is only 22%, and the 10-year overall survival rate is only 35% (Poon et al., 2002; Bosch et al., 2004; Marrero et al., 2018; Yang et al., 2019). It is critical, therefore, to identify sHCC patients with high risk of recurrence to initiate early intervention.

Wilms’ tumor 1-associating protein (WTAP) is a component of the mammalian *N* ^6^-methyladenosine (m6A) methyltransferase complex and acts by recruiting methyltransferase like 3 (METTL3) and methyltransferase like 14 (METTL14) into nuclear speckles (Ping et al., 2014). Increasing evidence shows that WTAP contributes to aggressive features in many tumors, including renal cell carcinoma, colorectal cancer, glioblastoma, and acute myeloid leukemia (AML) (Jin et al., 2012; Bansal et al., 2014; Zhang et al., 2016; Tang et al., 2018). So far, no analysis were done for the prognostic effects of WTAP in sHCC. Moreover, changes in paired liver adjacent non-neoplastic tissues (PANLT) have been found to predict survival (Hoshida et al., 2008; Utsunomiya et al., 2010; Utsunomiya et al., 2014). Whether the status of WTAP in liver tissues close to the primary tumor affects the outcomes of sHCC patients is unclear either.

The present study, focusing on a large cohort of Asian sHCC patients who received curative partial hepatectomy from a prospectively maintained database, aimed to clarify the prognostic value of WTAP levels in both tumor tissue and PANLT.

## Materials and methods

### Study population

This study was approved by the Institutional Medical Ethics Committee of Sun Yat-Sen University Cancer Center (SYSUCC), Guangzhou, China. The pathologically confirmed, non-metastatic sHCC between December 1998 and 2010 were obtained from the prospective created database. Only patients who underwent surgical resection, not ablation or transplantation, as the first course of therapy were included in the present study. The inclusion criteria were as followed: 1) solitary small HCC (≤3 cm); 2) no evidence of metastatic or residual disease; 3) presence of the HBV surface antigen; 4) primary and curative resection; 5) no metastatic or residual disease; 6) no preoperative adjuvant therapy; 7) complete clinical information and follow-up data.

### Follow-up

The evaluation and management approaches employed before surgical resection were as previously described (Villanueva, 2019). After curative partial hepatectomy, patients were examined at 1 month after resection and then generally at 3-month intervals in the first 2 years and every 3–6 months in subsequent years, until tumor recurrence was documented. Detailed information is presented in the Supplementary Materials.

### Outcomes and variable definitions

The primary endpoint was recurrence-free survival (RFS), which was defined as the time from the date of surgery to the date of first tumor recurrence (local or distant metastases identified by imaging technique and pathology). The secondary endpoint was overall survival (OS), which was defined as the time from the date of surgery to the date of death from any cause. Variable definitions were presented in the Supplementary Materials.

### Tissue microarray and immunohistochemistry

Detailed information of Tissue microarray and immunohistochemistry is presented in the Supplementary Materials.

### Statistical analysis

The optimal cutoff for WTAP expression levels was evaluated using maximally selected rank statistics (Lausen and Schumacher, 1992). Baseline characteristics in WTAP subgroups of PANLT were compared using Chi-square test in the case of two categorical factors, or ANOVA in the case of a categorical and a continuous factor. Correlations between continuous variables were assessed using the Spearman’s rank correlation coefficient. Survival rates were estimated using the Kaplan–Meier method with the log-rank test. The Cox regression model was utilized for multivariate survival analysis. Considering potential guarantee-time bias when the effects of the WTAP expression were evaluated for late recurrence, landmark analysis was adopted.

In order to confirm the robustness of analysis, the following three complementary approaches to adjust the comparison of survival rates among patients with low and high expression protein profile based on differences in baseline characteristics: the standard Cox proportional hazards regression model; the inverse probability of treatment weighting (IPTW) model; and the sensitivity analysis. Statistical analyses were performed using Stata version 16.0 (Stata Corp, College Station, TX) and R version 3.6.1 (R Foundation for Statistical Computing, Vienna, Austria). All statistical tests were two-sided, and *p* values less than 0.05 was considered significant.

In the multivariable analysis (MVA) with standard Cox proportional hazards regression model using the forward likelihood ratio method, we included all observed variables with clinical significance or a significant association with survival. Then, the interaction effect analysis was performed between the WTAP expression levels and other variables. The stratification analysis was further executed based on the significant interaction effects.

For the IPTW model analysis, each observation was weighted using the inverse probability of different group based on the propensity score. Then, a Cox proportional hazards model was fitted with the group as the only predictor variable. A robust sandwich variance estimator was used to account for the weighted nature of the sample. Prior work has verified that the IPTW allow for estimation of margin hazard ratios with minimal bias.

### Sensitivity analysis

Unknown or unmeasured variables (i.e., pre- or post- HBV DNA levels, post-relapse treatment and economic status) may also impact on the status of expression profile and the prognosis, although the IPTW analysis can address biases caused by observed variables. The sensitivity analysis, therefore, was performed for the unmeasured variables in the aforementioned analysis to measure their potential confounding effects on our results and to validate the robustness of the present analysis. We varied the prevalence of the elevated HBV DNA levels and the adjusted relapse HRs in the two different groups using estimates from prior studies of patients. Based on these studies studies (Chen et al., 2006; Xia et al., 2012; Kruse et al., 2014; Li et al., 2016), we assumed that the elevated HBV DNA levels of 10^5^ copy/ml or greater would be associated with an HR of 1.2–2. Using these data, we calculated adjusted HRs and 95% confidence intervals (CIs) for the low expression group.

## Results

### Patient characteristics and follow-up

The characteristics of the 307 sHCC patients, including 276 (89.9%) men and 31 (10.1%) women with a median age of 49 years (range, 26–78 years), are presented in Table 1 and Supplementary Table S1. Across the entire cohort, median tumor size was 2.0 cm (range, 0.8–3.0 cm). The median expression WTAP levels is 0 score (rang, 0–80) and 50 scores (range, 10–90) in sHCC and PANLT, respectively (Supplementary Figures S1A,B). With regard to ordinal variables, a weak negative correlation was observed between WTAP expression levels in sHCC and PANLT (r = −0.22, *p* < 0.001). No significant correlations were observed between clinical parameters and WTAP expression in either tumor or PANLT.

**TABLE 1:** Baseline patient characteristics according to expression profile of WTAP in PANLT^a^.

Characteristic	N = 307 (%)	No. (%) of patients		P
		Low expression cohort *n* = 121 (%)	High expression cohort *n* = 186 (%)	
Gender				0.212
Male	276 (89.9)	112 (92.6)	164 (88.2)	
Female	31 (10.1)	9 (7.4)	22 (11.8)	
Age, years				0.292
≤48	151 (49.2)	55 (45.5)	96 (51.6)	
>48	156 (50.8)	66 (54.5)	90 (48.4)	
AFP, ng/mL				0.995
≤25	132 (43.0)	52 (43.0)	80 (43.0)	
>25	175 (57.0)	69 (57.0)	106 (57.0)	
ALT, U/L				0.970
≤40	178 (58.0)	70 (57.9)	108 (58.1)	
>40	129 (42.0)	51 (42.1)	78 (41.9)	
Differentiation				0.975
Well	50 (16.3)	21 (17.4)	29 (15.6)	
Moderate	196 (63.8)	76 (62.8)	120 (64.5)	
Poor	54 (17.6)	21 (17.4)	33 (17.7)	
Undifferentiated	7 (2.3)	3 (2.5)	4 (2.2)	
Tumor size, cm				0.342
≤2	175 (57.0)	73 (60.3)	102 (54.8)	
>2	132 (43.0)	48 (39.7)	84 (45.2)	
Vascular invasion				0.822
Absent	238 (77.5)	93 (76.9)	145 (78.0)	
Present	69 (22.5)	28 (23.1)	41 (22.0)	
Envelope				0.429
Absent	191 (62.2)	72 (59.5)	119 (64.0)	
Present	116 (37.8)	49 (40.5)	67 (36.0)	
Liver cirrhosis				0.909
Absent	184 (59.9)	73 (60.3)	111 (59.7)	
Present	123 (40.1)	48 (39.7)	75 (40.3)	
Necrosis				0.145
Absent	168 (54.7)	60 (49.6)	108 (58.1)	
Present	139 (45.3)	61 (50.4)	78 (41.9)	
WTAP in tumor				0.004
Negative	185 (60.3)	61 (50.4)	124 (66.7)	
Positive	122 (39.7)	60 (49.6)	62 (33.3)	

^a^ PANLT, the paired adjacent non-neoplastic liver tissues.

In this cohort, 121 (39.4%) patients experienced recurrence, and 68 (22.1%) patients died during the follow-up period. The 5-year OS and RFS rates of the full cohort were 72.8% and 59.1%, respectively.

### Prognostic significance of Wilms’ tumor 1-associating protein expression

Both primary sHCC and PANLT were stained with a specific WTAP antibody, and clear and distinguishable brown staining of WTAP in the nucleus can be observed in the representative images (Supplementary Figure S2). Semiquantitative assessments were performed as described in the methods (Supplementary Materials).

**FIGURE 1:**
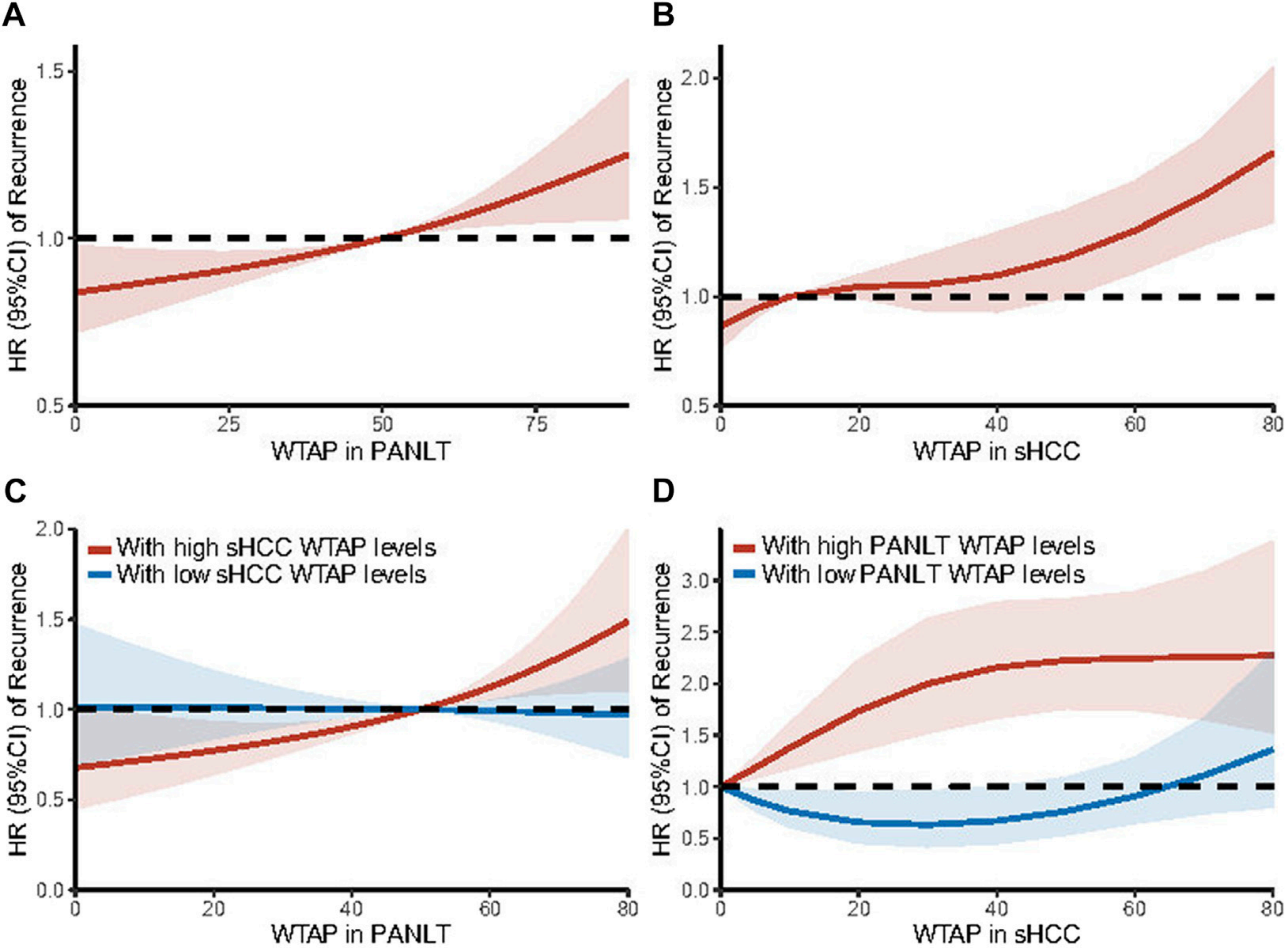
Trends in the adjusted HR of recurrence are illustrated according to WTAP expression levels in PANLT **(A)** and in sHCC **(B)** as a continuous variable by using restricted cubic splines with three knots. And based on interaction effects, stratification analyses were further executed in patients with high [**(C)**, red line] and low [**(C)**, blue line] sHCC WTAP levels for PANLT WTAP levels, and in patients with high [**(D)**, red line] and low [**(D)**, blue line] PANLT WTAP levels for sHCC WTAP levels. The restricted cubic spline allows for a non-linear relationship of WTAP expression levels with the HR of recurrence, estimated from the Cox model adjusted for all observational covariates. Results showed the HR of recurrence increased with elevated WTAP levels of PANLT and sHCC. Abbreviations: CI, confidence interval; HR, hazard ratio; PANLT, the paired adjacent non-neoplastic liver tissues; sHCC, small hepatocellular carcinoma.

Using maximally selected rank statistics, cutoff points were identified for high- and low-WTAP groups, with sHCC and PANLT levels analyzed independently, and is 5 scores and 50 scores, respectively (Supplementary Figures S1C,D). As part of sensitivity analysis, restricted cubic splines were executed to estimate effects on recurrence, with WTAP expression treated as an ordinal variable. The results confirmed that hazard risk of recurrence increased with high WTAP levels in sHCC and PANLT (Figures 1A,B).

In the unadjusted analysis, the following factors were identified as having significantly detrimental effects on recurrence: higher grade, larger tumor size, vascular invasion, necrosis, and elevated WTAP levels in non-neoplastic liver tissue. The 5-year RFS and OS rates for patients with high and low PANLT WTAP levels were 54.4% versus 66.7% and 69.4% versus 73.9%, respectively (Supplementary Figures S3A,C). No significant differences were observed in RFS or OS based on sHCC WTAP levels (Supplementary Figures S3B,D).

Results of multivariate analysis using the Cox proportional hazards model are shown in Table 2. After adjusting for all observational factors, RFS rates remained significantly different between groups with high and low WTAP in PANLT (HR 1.567, 95% CI 1.065–2.307, *p* = 0.023), while OS rates did not significantly differ (HR 1.524, 95% CI 0.912–2.545, *p* = 0.108).

**TABLE 2:** Multivariate analysis for recurrence-free survival and overall survival in small HCC patients.^a^

Characteristics (reference)	Recurrence-free survival		Overall survival	
	HR (95% CI)	P	HR (95% CI)	P
WTAP in PANLT (low levels)				
Cox model	1.567 (1.065–2.307)	0.023	1.524 (0.912–2.545)	0.108
IPTW model	1.584 (1.057–2.375)	0.026	1.485 (0.890–2.480)	0.130
WTAP in sHCC (low levels)	1.312 (0.897–1.920)	0.162	1.350 (0.816–2.233)	0.242
Gender (male)	0.570 (0.275–1.181)	0.130	0.746 (0.310–1.795)	0.513
Age (≤48 years)	1.331 (0.919–1.929)	0.131	0.999 (0.610–1.635)	0.996
AFP (≤20 ng/ml)	0.798 (0.546–1.168)	0.246	0.903 (0.537–1.518)	0.699
ALT (≤40 U/L)	1.336 (0.925–1.930)	0.122	1.135 (0.690–1.866)	0.618
Differentiation (moderate)^b^	1.619 (1.068–2.456)	0.023	0.966 (0.531–1.759)	0.910
Tumor size (≤2 cm)	1.486 (1.029–2.145)	0.035	1.759 (1.069–2.895)	0.026
Vascular invasion (absent)	1.382 (0.902–2.118)	0.137	2.652 (1.546–4.551)	<0.001
Envelope (absent)	1.023 (0.704–1.489)	0.904	1.121 (0.677–1.855)	0.658
Liver cirrhosis (absent)	1.270 (0.863–1.869)	0.225	1.503 (0.904–2.499)	0.116
Necrosis (absent)	1.756 (1.206–2.556)	0.003	2.003 (1.200–3.343)	0.008

^a^ To evaluate the effects of PANLT WTAP levels, the multivariate analysis were executed by both Cox and IPTW models adjusted for all covariates.

^b^ Due to relatively small number of patients for four subgroups, comparison was done in subgroup of poor and undifferentiated versus subgroup of well and moderate.

Abbreviations: CI, confidence interval; HR, hazard ratio; IPTW, the inverse probability of treatment weighting; PANLT, the paired adjacent non-neoplastic liver tissues; sHCC, small hepatocellular carcinoma.

### Inverse probability of treatment weighting analysis

To further confirm the effects of PANLT WTAP, we calculated weights for each case in threshold-based groups, and a propensity score with inverse probability of treatment weighting model was established to reevaluate HRs with variable weights and balance the observed effects. The results of the weighted model still indicate similarly significant outcomes (Table 2).

### Stratification analysis based on interaction effects

**FIGURE 2:**
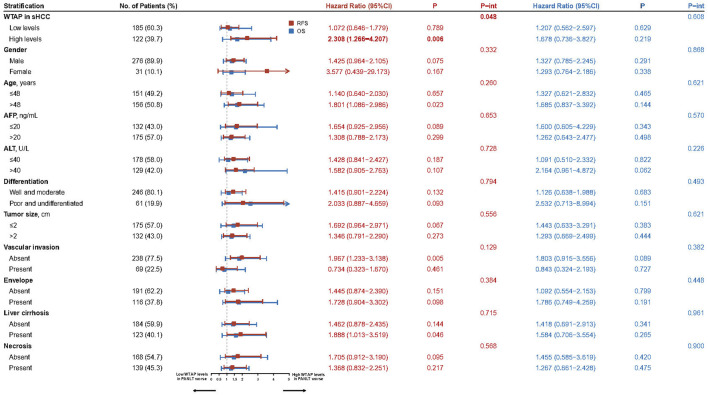
Forest plots for the interaction and stratification analyses based on the WTAP levels of PANLT and sHCC for RFS and OS. Abbreviations: CI, confidence interval; OS, overall survival; PANLT, the paired adjacent non-neoplastic liver tissues; RFS, recurrence-free survival; sHCC, small hepatocellular carcinoma.

Interaction effects between PANLT WTAP levels and other characteristics were examined using Cox regression analysis. One significant interaction was found between WTAP levels in nontumor tissue and WTAP levels in tumor tissue (*p* = 0.048; Figure 2). Stratification analysis revealed the following difference between the groups: relapse risk is more pronounced in patients with higher WTAP levels in both tumor and paired para-cancerous tissues.

The restricted cubic splines of hazard risk showed that, specifically for patients with high sHCC WTAP levels, recurrence risk increased with upregulated WTAP levels in PANLT (Figure 1C). Similarly, in patients with overexpression of WTAP in PANLT, recurrence risk was exacerbated by raised sHCC WTAP levels (Figure 1D).

In cohorts with high sHCC WTAP levels, there was a statistically significant difference in RFS between patients with high and low PANLT WTAP (HR 2.308, 95% CI 1.266–4.207; *p* = 0.006), whereas no such difference in RFS was found for the cohort with low sHCC WTAP expression (HR 1.072, 95% CI 0.646–1.779; *p* = 0.789; Figure 2; Supplementary Figure S4). Similarly, in cohorts with high PANLT WTAP expression, there was a statistically significant difference in RFS between patients with high and low sHCC WTAP (HR 1.872, 95% CI 1.164–3.008; *p* = 0.010), whereas no difference in RFS was found for the cohort with low PANLT WTAP expression (HR 0.754, 95% CI 0.393–1.448; *p* = 0.397; Supplementary Table S2).

### The prognostic role of Wilms’ tumor 1-associating protein expression for early and late recurrence

**FIGURE 3:**
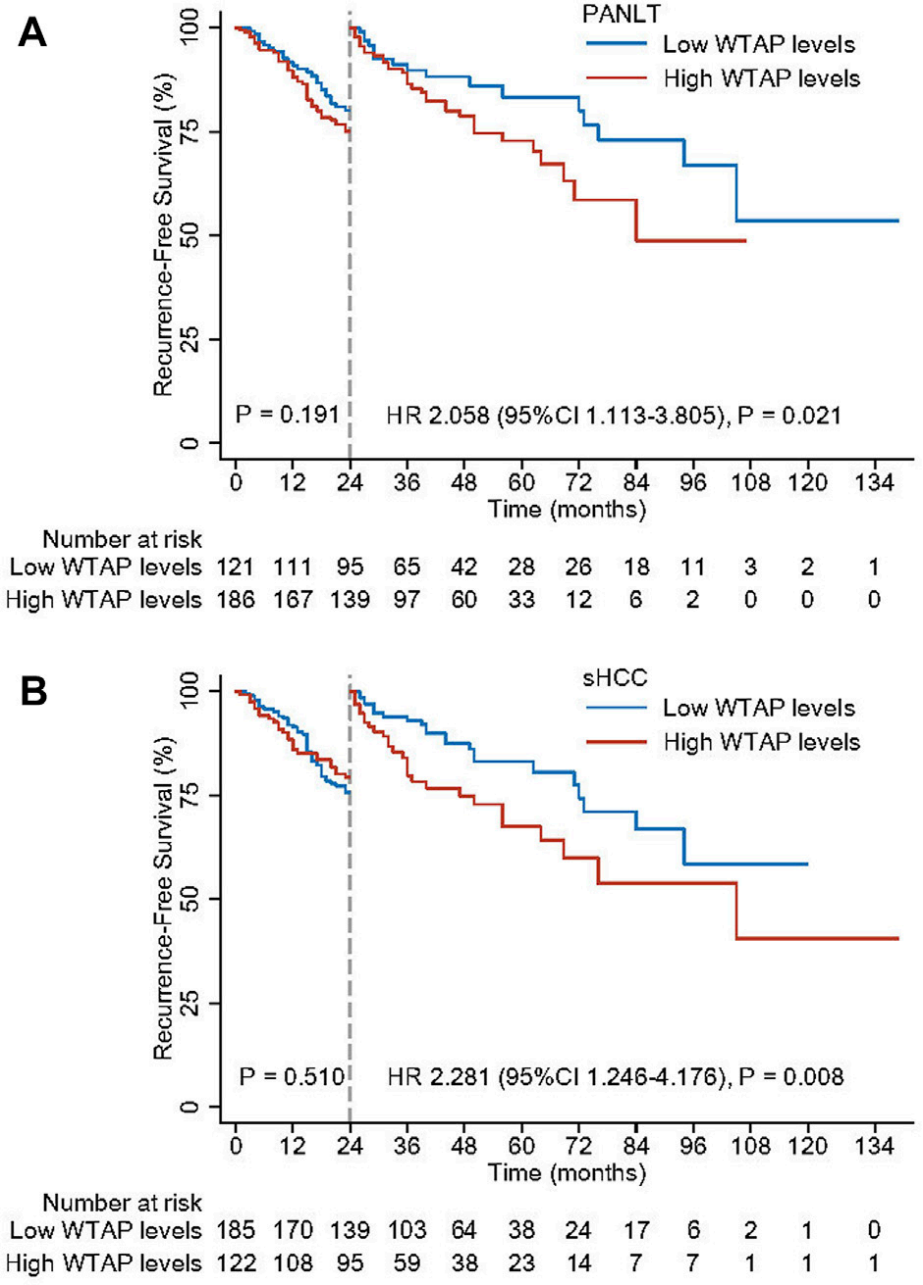
Landmark analyses with the mark point of 2 years for high and low WTAP levels in PANLT **(A)** and sHCC **(B)**. Abbreviations: CI, confidence interval; HR, hazard ratio; PANLT, the paired adjacent non-neoplastic liver tissues; sHCC, small hepatocellular carcinoma.

Based on the independent risk of recurrence associated with WTAP levels, specific effects on early and late recurrence were further explored. Using landmark analysis with a mark point of 2 years, significantly detrimental effects on late recurrence were observed for patients with high PANLT (HR 2.058, 95% CI 1.113–3.805; *p* = 0.021) and sHCC (HR 2.281, 95% CI 1.246–4.176; *p* = 0.008; Figure 3) WTAP levels. However, no significant differences were observed in early recurrence between low- and high-WTAP groups as defined by PANLT or sHCC.

### External extension analysis

In order to validate the effects of WTAP levels, two external cohorts (a Singapore cohort of 115 patients and a Shanghai cohort of 209 patients) were used (Roessler et al., 2012; Grinchuk et al., 2018). Based on evaluation of WTAP levels, which were treated as a continuous variable with restricted cubic splines, hazard risk of recurrence was shown to increase with elevated PANLT WTAP levels in both cohorts. With elevated HCC WTAP levels, increased HR of recurrence was observed in the Singapore cohort, but not in the Shanghai cohort (Supplementary Figure S5).

**FIGURE 4:**
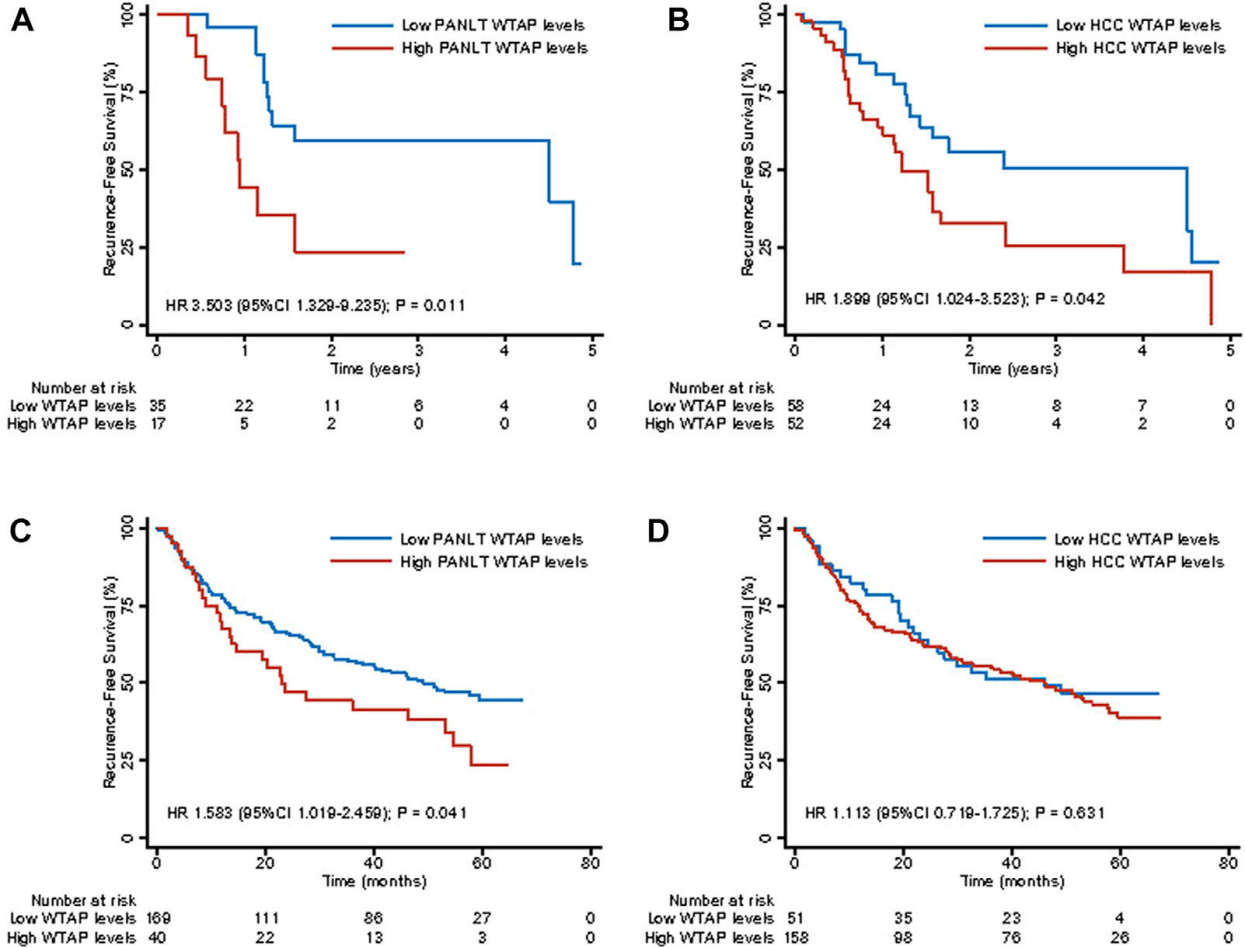
Kaplan-Meier survival curves for recurrence-free survival for high and low WTAP expression levels of liver tissue and HCC in external extension cohort of Singapore [**(A,B)**, respectively] and Shanghai [**(C,D)**, respectively]. Abbreviations: HCC, hepatocellular carcinoma.

Consistent with our analysis of patients with elevated PANLT WTAP levels, detrimental effects on RFS were confirmed in the Singapore cohort (HR 3.503, 95% CI 1.329–9.235; *p* = 0.011; Figure 4A) and the Shanghai cohort (HR 1.583, 95% CI 1.019–2.459; *p* = 0.041; Figure 4C), whereas no significant differences were observed in OS between high and low groups in the Singapore or Shanghai cohorts (HR 1.985, 95% CI 0.456–8.649, *p* = 0.361, and HR 1.645, 95% CI 0.990–2.734, *p* = 0.054, respectively; Supplementary Table S2). Meanwhile, for elevated HCC WTAP levels, detrimental effects on RFS were observed in the Singapore cohort (HR 1.899, 95% CI 1.024–3.523; *p* = 0.042; Figure 4B), but no significant differences were observed in the Shanghai cohort (HR 1.113, 95% CI 0.719–1.725; *p* = 0.631; Figure 4D). No significant differences in mortality were observed between the two cohorts (Supplementary Table S3).

### Sensitivity analysis

For patients with low PANLT WTAP levels, high WTAP levels were relatively robust in predicting detrimental relapse of patients with elevated HBV DNA levels (Table 3). For instance, assuming an HR of 1.2, elevated HBV DNA levels could not eliminate the significant detrimental effects of high WTAP levels, even if we assumed that none of the patients in the high group presented with elevated HBV DNA levels, and 100% of the patients in the low group presented with the elevated HBV DNA levels. However, assuming an HR of 2.2, elevated HBV DNA levels eliminated any significant difference between the two groups. This sensitivity analysis indicates that our analysis results were robust.

**TABLE 3:** Sensitivity analysis for hazard ratio of relapse adjusted for high HBV DNA levels in patients with high WTAP level in tumor tissue.

Prevalence of the elevated HBV DNA levels		HR		95% CI
Low levels^a^	High levels	High HBV DNA levels	High levels (adjusted for high HBV DNA levels)	
0.1	0.7	1.2	2.065	1.132–3.764
0.1	0.8	1.2	2.029	1.113–3.699
0	0.9	1.2	1.955	1.072–3.565
0	1	1.2	1.923	1.055–3.505
0.1	0.3	2.2	1.900	1.042–3.464
**0.1**	**0.4**	**2.2**	**1.746**	**0.958–3.183**

NOTE: Bold font indicates situations where high HBV DNA levels was strong enough to influence significance of rules (i.e., lower bound of 95% CI crossed 1). Values based on multivariate analysis in patients with high tumor WTAP levels adjusted relapse HR of 2.308 (95% CI, 1.266–4.207).

^a^ Low and High levels of WTAP in the non-neoplastic liver tissue.

Abbreviations: CI, confidence interval; HBV, Hepatitis B virus; HR, hazard ratio.

## Discussion

To the best of our knowledge, with respect to a large cohort with high prevalence of HBV infection, the present study is the first to show that increased PANLT WTAP levels independently predict risk of relapse in patients with HBV-positive sHCC. Interestingly, we found the PANLT WTAP levels could predict prognosis of sHCC patients, but sHCC WTAP levels could not. Based on interaction effects, we determined that patients with elevated PANLT WTAP expression and high sHCC WTAP levels had worse recurrence-free survival. Furthermore, landmark analysis showed the high PANLT WTAP and the high sHCC WTAP levels are related to increased risk of late recurrence. In addition, examination of external cohorts validated the detrimental effects of PANLT WTAP levels.

To prolong the survival time of individual sHCC patients, clinicians must identify sHCC patients at high risk for recurrence. Prior to now, numerous staging systems have been proposed, of which the Barcelona-Clinic Liver Cancer (BCLC) staging system is the most widely used in routine clinical practice. However, the prognosis of sHCC patients varies even within the same BCLC 0/A stage (Feng et al., 2012; Chan et al., 2018), implying that these systems are insufficient in their enumeration of anatomical and histopathological attributes, such as vascular invasion and tumor multinodularity, for patients who are increasingly diagnosed at earlier stages. Indeed, the main reason for these prognostic differences may be tumor heterogeneity as determined by epigenetic and genetic alterations. Unfortunately, using genome-wide expression profiling of primary HCC, Hoshida and colleagues failed to detect significant gene-expression changes associated with recurrence or mortality (Hoshida et al., 2008). Even though various studies have suggested biomarkers for HCC based on genetic alterations, tumor heterogeneity has limited their reproducibility and application (Lee et al., 2004; Cai et al., 2011; Nault et al., 2013; Zucman-Rossi et al., 2015; Nault et al., 2018; Muller et al., 2020). As previously reported, hepatocellular carcinomas are complex ecosystems in which tumor cells accumulate mutations and chromosomal aberrations and diverse nontumor cells are incorporated. For these reasons, it is challenging to identify and apply any single or molecular profile for clinical prediction of HCC patients.

Tumor diagnosis is based on morphological alterations observed in pathology. However, normal cells usually undergo a multi-step process of accumulating epigenetic and genetic alterations, turning into benign lesions, and ultimately ending up as a malignant tumor. The surrounding PANLT, for example, does not show morphological alterations, but does already exhibit substantial changes. Hoshida et al. (2008) found that gene-expression profiles of liver tissue adjacent to the resected tumor could be significantly correlated with prognosis. In addition, over recent decades, several studies have demonstrated that gene panels in non-neoplastic gastric mucosa, DNA methylation in non-neoplastic colorectal epithelium, microRNA expression profiles in biliary intraepithelial neoplasia and HBV DNA in non-neoplastic liver tissue can predict patient prognosis (Fujii et al., 2005; Tsuchiya et al., 2010; Utsunomiya et al., 2010; Utsunomiya et al., 2014; Wang et al., 2016; Grinchuk et al., 2018; Loeffler et al., 2020). In the present study, our results showed that, compared with patients with low WTAP levels in non-neoplastic liver tissue, patients with high PANLT WTAP levels have a significantly increased risk of recurrence, particularly late recurrence. These results are consistent with the model that late recurrence results from the arising of new primary tumors in a risk field, while early recurrence is the result of a locally invasive or incompletely resected tumor (Hoshida et al., 2008). In our cohort, sHCC WTAP levels could not predict RFS or OS.

With respect to the relationship between HCC WTAP levels and clinical prognosis, Chen et al. (2019) showed, in a cohort of only ninety HCC patients, that upregulated HCC WTAP expression is associated with poor HCC outcomes, including DFS and OS, although PANLT WTAP expression was not taken into consideration. However, analysis of numerous additional factors in a larger population with longer follow-up data failed to reveal significant differences in survival or recurrence based on tumor WTAP. These contradictory results may result from differences in patient populations, as our study is focused on patients with relatively homogeneous sHCC, while the previous study included cases of large HCC. Furthermore, data from two external cohorts (the Singapore cohort and the Shanghai cohort) were analyzed, and the adverse effects of HCC WTAP were only observed for the Singapore cohort, where they were detected for recurrence, but not OS. Moreover, consistent with our conclusions from the sHCC cohort, the previously underappreciated role of PANLT WTAP in HCC recurrence was convincingly demonstrated in these two external cohorts. In addition, based on interaction effects, stratification analysis showed that high PANLT WTAP levels affect recurrence particularly adversely in those with increased sHCC WTAP levels. These analyses indicate that WTAP expression, not only in tumors but also in PANLT, should be considered to evaluate patients in a more comprehensive and accurate manner.

The present study has several limitations. The first limitation is its single-institute nature. Although we performed multifaceted analyses to corroborate the robustness of our results, including external extension analysis, similarly scaled studies remain necessary to further validate our results. Secondly, our cohort, composed of patients with HBV-positive Asian sHCC, was relatively homogenous. Our conclusions must be further confirmed in HCC cohorts, although the external validation of HCC with sequencing data has been done. To be noticeable, the data we used is transcriptomic data from the two cohorts, which differs from the current study using protein expression, and this partial analysis in two external cohorts is extension analysis nature for the finding. Lastly, the mechanism by which WTAP levels in PANLT impact relapse of small HCC was not revealed in the present study. The biological function of WTAP requires further investigation.

In summary, we herein present the first comprehensive clinical analysis utilizing a large prospective HBV-positive cohort, revealing the importance on recurrence of WTAP in both sHCC and PANLT. High levels of PANLT WTAP independently predict sHCC recurrence, particularly when accompanied by sHCC WTAP. Moreover, our study emphasizes that both tumor tissues and PANLT need to be considered together in future investigations to obtain a more comprehensive and accurate evaluation for recurrence risk.

## Data Availability

All data generated or analyzed during this study are included in this article and its online Supplementary Material files. All data in our study have been recorded at Sun Yat-sen University Cancer Center with a Research Data Deposit (No. 2209160002). Further inquiries can be directed to the corresponding author.

## Supplementary material

SUPPLEMENTARY FIGURE S1 The distribution of WTAP expression in sHCC **(A)** and PANLT **(B)**, respectively. The definition of cutoff for WTAP expression in sHCC **(C)** and PANLT **(D)** based on maximally selected rank statistics. Abbreviations: sHCC, small hepatocellular carcinoma; PANLT, the paired adjacent non-neoplastic liver tissues.

SUPPLEMENTARY FIGURE S2 Immunohistochemistry staining for WTAP expression in PANLT and sHCC. High WTAP expression levels in PANLT and low WTAP in sHCC for Case #1; high WTAP in PANLT and sHCC for Case #2; low WTAP in PANLT and high WTAP in sHCC for Case #3. Abbreviations: PANLT, the paired adjacent non-neoplastic liver tissues; sHCC, small hepatocellular carcinoma.

SUPPLEMENTARY FIGURE S3 Kaplan-Meier survival curves for recurrence-free survival and overall survival for high and low WTAP expression levels in PANLT **(A,C)** and sHCC **(B,D)**. Abbreviations: CI, confidence interval; HR, hazard ratio; PANLT, the paired adjacent non-neoplastic liver tissues; sHCC, small hepatocellular carcinoma.

SUPPLEMENTARY FIGURE S4 Based on interaction effects, comparison of recurrence-free survival between high and low PANLT WTAP levels were analyzed in patients with high **(A)** and low **(B)** sHCC WTAP levels. Abbreviations: CI, confidence interval; HR, hazard ratio; PANLT, the paired adjacent non-neoplastic liver tissues; sHCC, small hepatocellular carcinoma.

SUPPLEMENTARY FIGURE S5 Trends in the hazard ratio of recurrence are illustrated according to the PANLT and HCC WTAP expression levels as a continuous variable by using restricted cubic splines with three knots in external validation cohort of Singapore **(A,B)** and Shanghai **(C,D)**. Abbreviations: CI, confidence interval; HR, hazard ratio; HCC, hepatocellular carcinoma.

## Abbreviations

HCC, Hepatocellular carcinoma; WTAP, Wilms’ tumor 1-associating protein; RFS, Recurrence-free survival; PANLT, Paired adjacent non-neoplastic liver tissues; HBV, Hepatitis B virus; sHCC, Small hepatocellular carcinoma; HR, Hazard ratio; CI, Confidential interval; m6A, N6-methyladenosine; METTL3, Methyltransferase like 3; METTL14, Methyltransferase like 14; AML, Acute myeloid leukemia; SYSUCC, Sun Yat-Sen University Cancer Center; OS, Overall survival; BCLC, Barcelona-Clinic Liver Cancer.
