# Supplementary material for: Impact of WTAP in small HCC and paired adjacent non-neoplastic liver tissue on recurrence: A cohort study with external extension analysis
Source: Front Cell Dev Biol. 2022 Nov 7;10:973548. doi: 10.3389/fcell.2022.973548 (PMC9676468; doi:10.3389/fcell.2022.973548)

**Supplementary Materials *For***

**Impact of** **WTAP in** **small** **HCC and paired adjacent non-neoplastic liver tissue on** **recurrence: a cohort study with** **external extension analysis**

**Author**

Jin-Ling Duan^a,b #^, Min-Hua Deng^a,c #^, Zhi-Cheng Xiang^a,b^, Jin-Long Hu^a,b^, Chun-Hua Qu^b^, Tian-Chen Zhu^a,b^, Ming-Xing Xu^d^, Jie-Wei Chen^b^, Juan-Juan Xie^a^, Dan Xie^a,b^, Mu-Yan Cai^a,b^*, Mei Li^b^*, Hu Liang^a,e^*

**Supplementary Materials and Methods**

Study population

Follow-up

Outcomes and variable definitions

Tissue microarrays and immunohistochemistry

External extension analysis

**Supplementary Tables**

Table S1.

Table S2.

Table S3.

**Supplementary Figures**

Fig. S1

Fig. S2

Fig. S3

Fig. S4

Fig. S5

**Supplementary Materials and Methods**

**Study population**

This study was approved by the Institutional Medical Ethics Committee of Sun Yat-Sen University Cancer Center (SYSUCC), Guangzhou, China. The pathologically confirmed, non-metastatic sHCC between December 1998 and 2010 were obtained from the prospective created database. Written informed consent for the use of clinical data and collected samples for future studies was obtained when the patients were admitted to receive treatment as a general standard procedure for patients treated in our centre. All patient records were anonymous and de-identified before the analysis.

In order to validate the effects of WTAP levels, two publicly available data with the gene expression profiling, including a Singapore cohort of 115 patients ^1^ and a Shanghai cohort of 209 patients ^2^, were used. These two external cohorts are publicly available at GEO: GSE76427 and GSE14520 for Singapore cohort and Shanghai cohort, respectively.

**Follow-up**

The evaluation and management approaches employed before surgical resection were as previously described ^3^. After curative partial hepatectomy, patients were examined with abdominal ultrasonography, chest radiography, and laboratory tests and serum AFP analysis. These assessments were performed 1 month after resection and then generally at 3-month intervals in the first 2 years and every 3-6 months in subsequent years, until tumor recurrence was documented. In cases where tumor recurrence was suspected, further tests, including computed tomography (CT) and magnetic resonance imaging (MRI), were performed, and biopsies were employed if necessary.

**Outcomes and variable definitions**

The primary endpoint was recurrence free survival (RFS), which was defined as the time from the date of surgery to the date of first tumor recurrence (local or distant metastases identified by imaging technique and pathology). The secondary endpoint was overall survival (OS), which was defined as the time from the date of surgery to the date of death from any cause. The extent of tumor differentiation was determined based on the criteria proposed by Edmonson and Steiner. Vascular invasion in each HCC specimen was identified in several serial cross sections. Patients who had macroscopic and/or microscopic tumor emboli within the large capsular vessels, the central hepatic vein, or the portal vein were considered to have vascular invasion. Cirrhosis was defined by the presence of fibrous septa throughout the liver that subdivided the parenchyma into nodules.

**Tissue microarrays and immunohistochemistry**

Tissue microarrays (TMAs) were assembled in accordance with our previous methodology ^4^. Triplicate 0.6 mm diameter cylindrical areas were punched from representative regions of an individual donor tissue block and re-embedded into a recipient paraffin block in a defined position using a tissue arraying instrument (Beecher Instruments, Silver Spring, MD).

The primary antibody was first tested and optimized on whole-tissue sections and test arrays. Negative control was set without implying the primary antibody. Once an appropriate dilution had been determined, a set of TMAs containing all samples with triplicate sHCC and PANLT of each case were stained for WTAP, using standard two-step indirect immunohistochemistry. Deparaffinization was performed using xylene and then the sections were rehydrated with graded ethanol. Slides were then placed in target retrieval solution (EDTA, pH 9.0) and heated for 2.5 min in a pressure cooker at boiling temperature. After cooling for 30 min, endogenous peroxidase activity was blocked by treatment with 3% hydrogen peroxide for 15 min. The sections were washed with Tris-buffered saline. Primary mouse anti-WTAP monoclonal antibodies with dilution at 1:1000 (Proteintech, 60188-1-Ig) were applied for 1 hour at room temperature. Detection was accomplished with the Dako Envision System, followed by chromogen detection with diaminobenzidine (DAB). The sections were counterstained with hematoxylin and mounted.

Semiquantitative assessments for WTAP expression in each case were scored by the percentage of cells with positive nuclear staining over the total number of cells in triplicated fields. Scores were assigned by using 5% increments (0%, 5%, 10% . . . 100%). The evaluations were performed independently by two experienced pathologists (J-L Hu and J-W Chen), who were blinded to all clinicopathologic and outcome variables. Individual scores were averaged and in cases of difference by more than 20%, a third pathologist (M-Y Cai) was consulted and provided an appropriate, agreed-upon score.

**External extension analysis**

Two external extension cohorts were analyzed in our study, including the Singapore cohort of 115 patients and the Shanghai cohort of 209 patients. For evaluating the prognostic value of WTAP levels, multivariable Cox regression model using the forward likelihood ratio method was executed in two cohorts to adjust for covariables, including demographic information and tumor information, such as age, gender, TNM system, ALT, cirrhosis status, multinodular, AFP, or HBV status. To be noticeable here, firstly, the data we used is transcriptomic data from the two cohorts, which differs from the current study using protein expression. Secondly, the Shanghai cohort comprises HBV-positive (97%) HCC patients, and the Singapore cohort included HCC patients with mixed etiologies, and we cannot obtain information of sHCC patients included in these cohorts. Thirdly, TNM stage is only one factor included in multivariable model but all other important covariables, such as multinodular, cirrhosis and AFP, were excluded, so multivariable analysis was done without TNM stage in Shanghai cohort. In Singapore cohort, WTAP expression does be an independent prognostic factor when adjusted for all supplied clinical factors including age, gender and TNM stage. Above all, this partial analysis in two external cohorts is extension analysis nature.

**REFERENCE**

1. Grinchuk, O.V., et al., *Tumor-adjacent tissue co-expression profile analysis reveals pro-oncogenic ribosomal gene signature for prognosis of resectable hepatocellular carcinoma.* Mol Oncol, 2018. **12**(1): p. 89-113.

2. Roessler, S., et al., *Integrative genomic identification of genes on 8p associated with hepatocellular carcinoma progression and patient survival.* Gastroenterology, 2012. **142**(4): p. 957-966 e12.

3. Villanueva, A., *Hepatocellular Carcinoma.* N Engl J Med, 2019. **380**(15): p. 1450-1462.

4. Cai, M.Y., et al., *EZH2 protein: a promising immunomarker for the detection of hepatocellular carcinomas in liver needle biopsies.* Gut, 2011. **60**(7): p. 967-976.

**Table S1. Baseline patient characteristics according to expression profile of WTAP in sHCC ^a^.**

|  |  | No. (%) of patients | |  |
| --- | --- | --- | --- | --- |
| Characteristic | N = 307 (%) | Low expression cohort  n = 185 (%) | High expression cohort  n = 122 (%) | P |
| Gender |  |  |  | 0.299 |
| Male | 276 (89.9) | 169 (91.4) | 107 (87.7) |  |
| Female | 31 (10.1) | 16 (8.6) | 15 (12.3) |  |
| Age, years |  |  |  | 0.162 |
| ≤48 | 151 (49.2) | 85 (45.9) | 66 (54.1) |  |
| >48 | 156 (50.8) | 100 (54.1) | 56 (45.9) |  |
| AFP, ng/mL |  |  |  | 0.732 |
| ≤25 | 132 (43.0) | 81 (43.8) | 51 (41.8) |  |
| >25 | 175 (57.0) | 104 (56.2) | 71 (58.2) |  |
| ALT, U/L |  |  |  | 0.441 |
| ≤40 | 178 (58.0) | 104 (56.2) | 74 (60.7) |  |
| >40 | 129 (42.0) | 81 (43.8) | 48 (39.3) |  |
| Differentiation |  |  |  | 0.841 |
| Well | 50 (16.3) | 33 (17.8) | 17 (13.9) |  |
| Moderate | 196 (63.8) | 116 (62.7) | 80 (65.6) |  |
| Poor | 54 (17.6) | 32 (17.3) | 22 (18.0) |  |
| Undifferentiated | 7 (2.3) | 4 (2.2) | 3 (2.5) |  |
| Tumor size, cm |  |  |  | 0.416 |
| ≤2 | 175 (57.0) | 102 (55.1) | 73 (59.8) |  |
| >2 | 132 (43.0) | 83 (44.9) | 49 (40.2) |  |
| Vascular invasion |  |  |  | 0.907 |
| Absent | 238 (77.5) | 143 (77.3) | 95 (77.9) |  |
| Present | 69 (22.5) | 42 (22.7) | 27 (22.1) |  |
| Envelope |  |  |  | 0.828 |
| Absent | 191 (62.2) | 116 (62.7) | 75 (61.5) |  |
| Present | 116 (37.8) | 69 (37.3) | 47 (38.5) |  |
| Liver cirrhosis |  |  |  | 0.102 |
| Absent | 184 (59.9) | 104 (56.2) | 80 (65.6) |  |
| Present | 123 (40.1) | 81 (43.8) | 42 (34.4) |  |
| Necrosis |  |  |  | 0.265 |
| Absent | 168 (54.7) | 106 (57.3) | 62 (50.8) |  |
| Present | 139 (45.3) | 79 (42.7) | 60 (49.2) |  |
| WTAP in PANLT ^b^ |  |  |  | 0.004 |
| Low | 121 (39.4) | 61 (33.0) | 60 (49.2) |  |
| High | 186 (60.6) | 124 (67.0) | 62 (50.8) |  |

Abbreviations: sHCC, small hepatocellular carcinoma; PANLT, the paired adjacent non-neoplastic liver tissues.

a The cutoff value of WTAP in sHCC is 5 scores.

b The cutoff value of WTAP in PANLT is 50 scores.

**Table S2. Survival analysis in patients with high versus low sHCC WTAP levels based on interaction analysis with PANLT WTAP levels.**

|  | Recurrence-free survival | |  |  | Overall survival | |  |
| --- | --- | --- | --- | --- | --- | --- | --- |
|  | HR (95% CI) | P | P-int |  | HR (95% CI) | P | P-int |
| **PANLT WTAP levels** |  |  | 0.048 |  |  |  | 0.608 |
| All patients | 1.312 (0.897-1.920) | 0.162 |  |  | 1.591 (0.945-2.678) | 0.081 |  |
| Low levels | 0.754 (0.393-1.448) | 0.397 |  |  | 1.101 (0.474-2.555) | 0.823 |  |
| High levels | 1.872 (1.164-3.008) | 0.010 |  |  | 1.480 (0.826-2.653) | 0.188 |  |

Abbreviations: CI, confidence interval; HR, hazard ratio; PANLT, the paired adjacent non-neoplastic liver tissues; sHCC, small hepatocellular carcinoma.

**Table S3. Multivariable analyses in external validation cohorts.**

|  | Recurrence-free survival | |  | Overall survival | |
| --- | --- | --- | --- | --- | --- |
| High versus low levels | HR (95% CI) | P |  | HR (95% CI) | P |
| **Singapore cohort** |  |  |  |  |  |
| PANLT WTAP | 3.503 (1.329-9.235) | 0.011 |  | 1.985 (0.456-8.649) | 0.361 |
| HCC WTAP | 1.899 (1.024-3.523) | 0.042 |  | 1.033 (0.447-2.385) | 0.940 |
| **Shanghai cohort** |  |  |  |  |  |
| PANLT WTAP | 1.583 (1.019-2.459) | 0.041 |  | 1.645 (0.990-2.734) | 0.054 |
| HCC WTAP | 1.113 (0.719-1.725) | 0.631 |  | 1.143 (0.675-1.935) | 0.618 |

Abbreviations: CI, confidence interval; HR, hazard ratio; PANLT, the paired adjacent non-neoplastic liver tissues; HCC, hepatocellular carcinoma.

**Fig. S1**


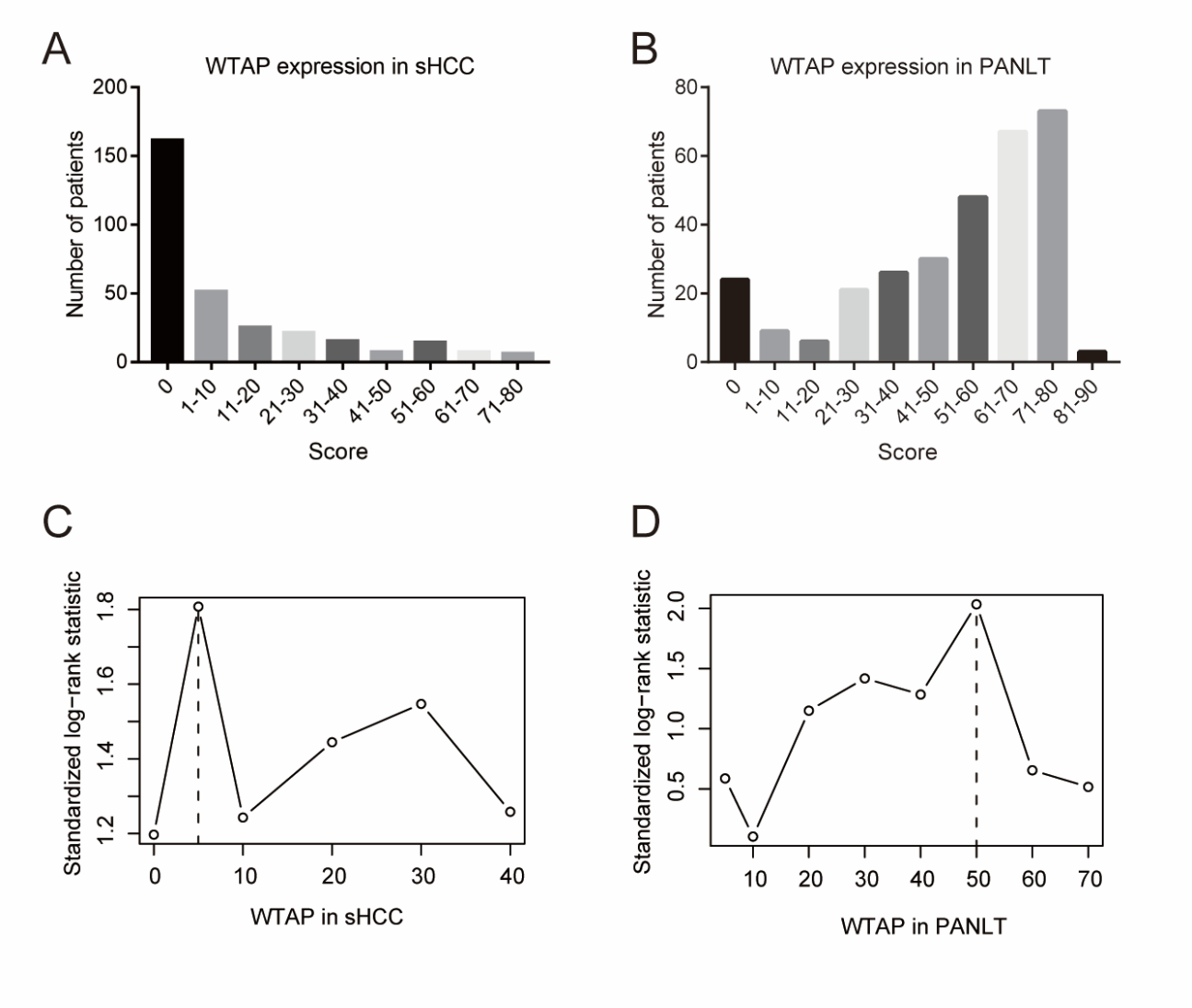


**Fig. S2**


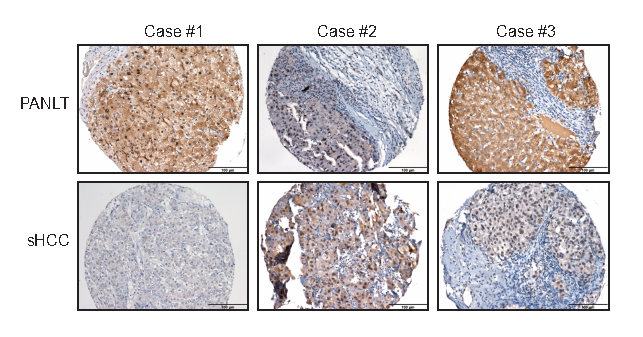


**Fig. S3**


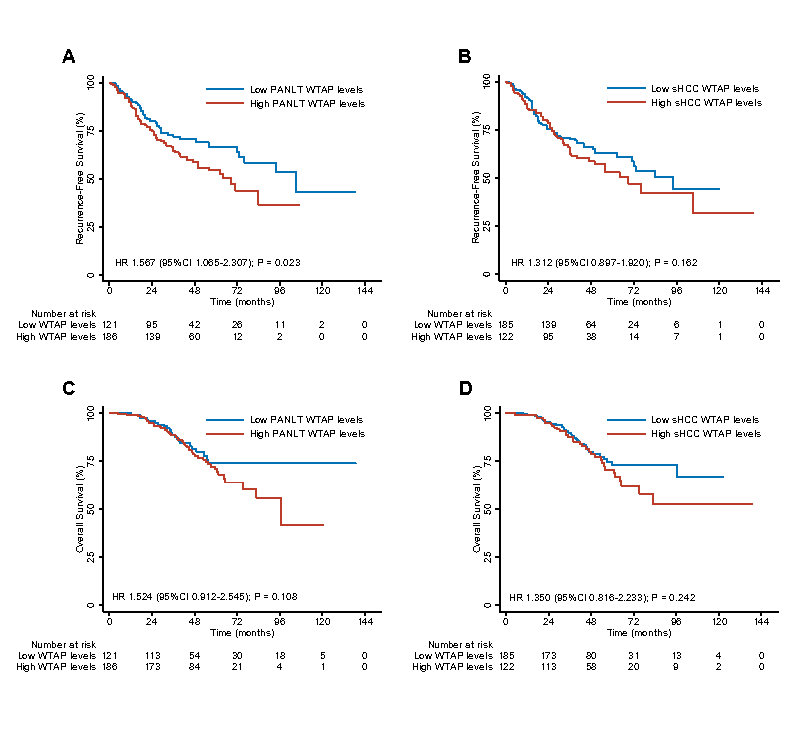


**Fig. S4**


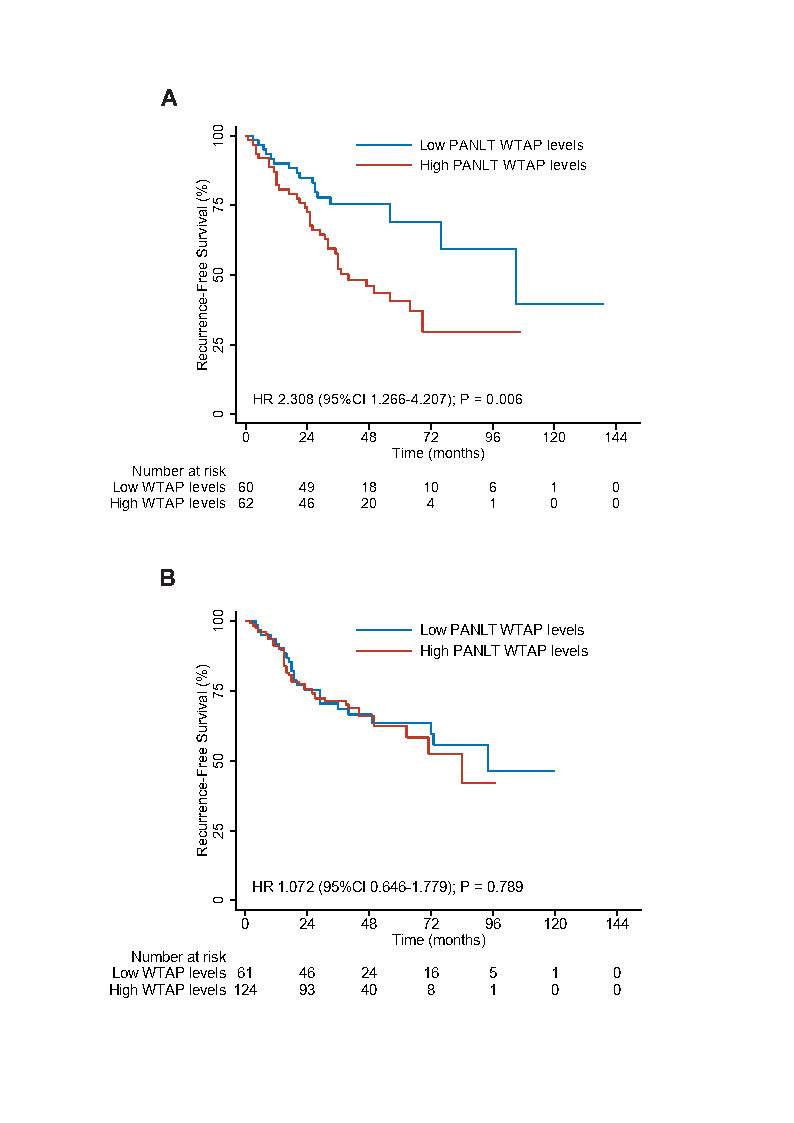


**Fig. S5**


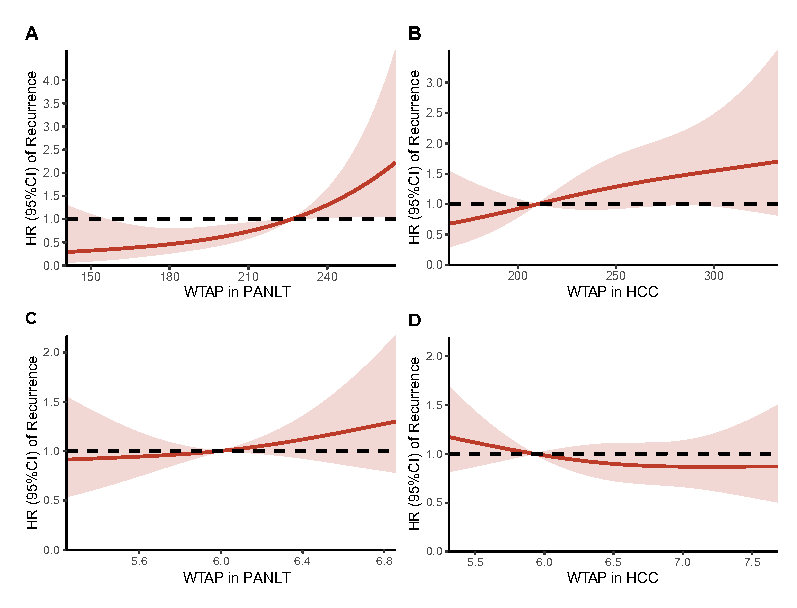

Supplement: Supplementary file 1 [file DataSheet1.docx]
